# Quality of life in women who were exposed to domestic violence during pregnancy

**DOI:** 10.1186/s12884-016-0810-6

**Published:** 2016-01-26

**Authors:** Zahra Tavoli, Azadeh Tavoli, Razieh Amirpour, Reihaneh Hosseini, Ali Montazeri

**Affiliations:** Department of Gynecology and Obstetrics, Ziaeian Hospital, School of Medicine, International Campus, Tehran University of Medical Sciences, Tehran, Iran; Department of Gynecology and Obstetrics, School of Medicine, Lorestan University of Medical Sciences, Lorestan, Iran; Department of Psychology, Faculty of Humanity Studies, Tarbiat Modares University, Tehran, Iran; School of Medicine, Lorestan University of Medical Sciences, Lorestan, Iran; Department of Gynecology and Obstetrics, Arash Hospital, Tehran University of Medical Sciences, Tehran, Iran; Mental Health Research Group, Health Metrics Research Center, Iranian Institute for Health Sciences Research, ACECR, Tehran, Iran; Faculty of Humanity Sciences, University of Science & Culture, ACECR, Tehran, Iran

**Keywords:** Quality of life, Domestic violence, Pregnant women

## Abstract

**Background:**

Quality of life in pregnant women is an important issue both for women’s and fetus’ health. This study aimed to examine quality of life in a group of women who were exposed to domestic violence during pregnancy.

**Methods:**

This was a cross sectional study of quality of life among a consecutive sample of pregnant women attending to a teaching hospital in Lorestan, Iran. Women were screened for experiencing violence using the Abuse Assessment Screen (AAS) questionnaire and were categorized as psychological abused, physical abused and non-abused groups. Quality of life was assessed using the Short-Form 36 Health Survey (SF-36). One-way analysis of variance and t-test were used to examine differences in quality of life in the study sub-samples. In addition logistic regression analyses were performed to investigate the association between general health and mental health and independent variables including age, education, parity and type of violence.

**Results:**

In all 266 pregnant women were approached, of which 230 (86.5 %) agreed to participate in the study. Of these, 149 women (64.8 %) reported that they had experienced either physical or psychological violence during pregnancy. A significant difference between abused and non-abused groups was identified, with the abused group recording lower mean scores on all sub-scales with the exception of the bodily pain (*p* = 0.27). In addition comparing quality of life between physical and psychological abused groups, women who reported physical violence recorded lower mean scores for physical functioning, role physical, bodily pain and general health, while women reporting psychological abuse had lower mean scores on social functioning, role emotional, vitality and mental health. Comparison between the physically and psychologically abused groups indicated significant differences only for role physical (*p* = 0.04), bodily pain (*p* = 0.003) and general health (*p* = 0.04). After adjusting for age, parity, and education, physical abuse was associated with poor physical health (OR = 2.13, 95 % CI = 1.05–4.36, *p* = 0.03), while emotional abuse was significantly associated with poor mental health (OR = 1.89, 95 % CI = 1.09–3.84, *p* = 0.04).

**Conclusion:**

Domestic violence against women during pregnancy in Iran was evident and this had significant adverse association with their quality of life. Indeed health care professionals involved in the care of women need to be aware of the extent of the problem and consider how it may be impacting on the women in their care.

## Background

Domestic violence, known as the most common type of gender-related violence, is of particular social and health concern [1]. Domestic violence against women encompasses any physical, sexual or emotional abuses imposed upon women in family relationships [2, 3]. A recent comprehensive review published by the World Health Organization in 2013 reported that ‘the global prevalence of physical and/or sexual intimate partner violence among all ever-partnered women was 30 %. The prevalence was highest in the WHO African, Eastern Mediterranean and South-East Asia Regions, where approximately 37 % of ever-partnered women reported having experienced physical and/or sexual intimate partner violence at some point in their lives’ (a total of 185 studies from 86 countries included and data from 155 studies in 81 countries provided the estimates) [4]. The review reported the following health effects of intimate partner violence: HIV and other sexually transmitted infections, induced abortion, low birth weight and prematurity, harmful alcohol use, depression and suicide, non-fatal injuries, and fatal injuries.

There is evidence that women may be more vulnerable to abuse during pregnancy and the postpartum period. As such it is argued that pregnancy not only does not provides security from intimate partner violence but also increases the risk of abusive relationships [5, 6]. A systematic review of the literature on violence against pregnant women in developing countries found that prevalence of violence among pregnant women ranged from 4 % to 29 % and the main risk factors for abuse were low-income, low education in both partners, and unplanned pregnancy [7]. The review included 6 studies from India, Pakistan, China and Ethiopia. All studies were cross-sectional in nature and all together studied 120421 pregnant women.

There is evidence that violence against pregnant women in Iran is high. For instance a study of 313 pregnant women found that 55.9 % of women had experienced violence during pregnancy including psychological violence (43.5 %), physical violence (10.2 %), and sexual violence (17.2 %) [8]. Also, it has been shown that violence against pregnant women might differ in different geographical areas in Iran. A study from West Azerbaijan Province with a sample of 1300 pregnant women aged 18–39 years found that 72.8 % of women reported that they had experienced IPV during their last pregnancy [9]. A recent study from Mazandaran Province (Northern Iran) studying 301 pregnant women aged 15–45 years found that 34.5 % of pregnant women had experienced psychological violence, 28.2 % physical violence, and 3.6 % sexual violence [10]. It seems that such observations reflect the fact that firstly violence against pregnant women in Iran is not confined to a defined geographical area and is prevalent through the country, and secondly depending on cultural differences that exist in different parts of the country (mostly related to gender role outlook), there might be some differences in prevalence of abuse.

Violence against pregnant women has several severe adverse effects not only on women’s health but also might harm the fetus. Several studies reported adverse outcomes including increase in fetal injury, perinatal death (prenatal death and early neonatal death), preterm birth, low birth weight, miscarriage, placental abruption, premature rupture of membranes, rupture of urethra, bleeding, prenatal hospitalization, infection, and adverse mental health consequences and maternal behavioral risks to perinatal outcomes including depression, anxiety disorders, post-traumatic stress disorder, suicide (attempts), delayed entry into prenatal care, poor maternal nutrition and use of tobacco, alcohol [11–18].

A study examining types of abuse compared physical, psychological and sexual violence among samples of pregnant women and found that the psychological abused group had a higher risk of postnatal depression compared with non-abused group. They were also at a higher risk of thinking about self-harm and had significantly poorer mental health-related quality of life. Although unusual, the higher risks of postnatal depression and self-harm were not evident in the physical and/or sexual abused group [19]. Thus it is not surprising if one believes that quality of life in pregnant women who are suffering from violence is a very important issue. Although recently the literature on violence against women during pregnancy is growing both from developed and developing countries [20–26], studies on relationship between domestic violence and quality of life of abused women during and after pregnancy are scarce.

There are no published papers examining quality of life in Iranian women during pregnancy. The available studies examining quality of life in abused pregnant women suggest that the intimate partner abuse has short-term and long-term negative health consequences [27, 28]. In a study comparing quality of life among four groups of women including pregnant women, it was found that the baseline quality of life of the victims of intimate partner violence was significantly impaired compared with the non-abused controls [29].

In Iran the majority of women attend free antenatal care at their local, state, or teaching hospitals. During their pregnancy generally attend a minimum of 12 visits. There is no universal postnatal care provided to but women can attend the hospital or visit doctor if she or the baby are experiencing physical health issues. Husbands usually do not attend antenatal or postnatal appointments. The focus of the antenatal care is on the obstetric health of mother and baby. Little consideration is given to social factors that may also impact on mother’s and baby’s health such as physical or emotional abuse from a husband. Therefore, this study aimed to identify what proportion of pregnant women attending a large teaching hospital were experiencing physical or psychological abuse by their husbands and the impact of this abuse on their quality of life during pregnancy. To the best of our knowledge this study is among a few existing literature that focus on health-related quality of life in pregnant women who had experienced violence. It was hoped that the findings from this study might contribute to the literature on the topic and perhaps provide evidence for developing appropriate services and practical therapeutic programs in health care centers and clinical settings.

## Methods

### Design and data collection

This was a cross-sectional study of quality of life among a consecutive sample of pregnant women attending to a general teaching hospital affiliated to Lorestan University of Medical Sciences, Lorestan, Iran during a complete calendar year from March 2012 to March 2013. All women attending antenatal care at the hospital who were at their last trimester were asked to participate in the research study by the main investigator (ZT). Women were approached in the waiting room. Participation was voluntary and would not impact in any way on the women’s antenatal care. Women completed a short face-to-face interview with the investigator in which she was asked about a history of psychological disorders and administered the study questionnaires. Responses were securely recorded on a laptop computer and were only accessible by the senior investigator. Women were excluded from the study (not invited to complete the study questionnaires) if they had history of psychological disorders, physical morbidity, and drug addiction.

### Study questionnaires

#### Abuse Assessment Screen (AAS)

It was used to screen the domestic violence. It contains 5 questions and identifies if a woman is experiencing intimate violence. One item specifically indicates if a pregnant woman have been slapped, kicked or physically hurt by someone [30]. Women were assigned to the psychological abused group if indicated that they have not been slapped, kicked or physically hurt but their partner used offensive language, kept them from going to see family, relatives and friends, or abused them emotionally etc. Accordingly women were grouped into three sub-samples: those who experienced physical violence, those who experienced psychological violence, and those who did not experience violence.

#### Short Form Health Survey (SF-36)

We used the Short Form Health Survey (SF-36) as outcome measure. The psychometric properties of the Iranian version of SF-36 are well documented [31]. The SF-36 contains 8 subscales assessing physical functioning, role physical, bodily pain, general health, vitality, social functioning, role emotional, and mental health. Possible score on each subscale range from 0 (the worse) to 100 (the best) conditions.

### Statistical analysis

The descriptive statistics including frequency, and mean (SD) was used to explore the data. We used t-test and one-way analysis of variance (ANOVA) and post hoc tests (Tukey HSD test) for comparing quality of life scores among the study sub-groups. The categorical data were compared using the chi-square. Logistic regression analysis was performed to examine the association between general health and mental health as outcome variables and age, education, parity and type of violence as independent variables. For the purpose of the analysis the general health and the mental health scores were categorized as equal or grater than mean (desired outcome) and less than mean (poor outcome). Data were analyzed using SPSS software.

### Ethics

The ethics committee of Lorestan University of Medical Sciences, Lorestan, Iran approved the study. All participants gave their written informed consent. We ensured all women that their information would be kept confidential. No one in the hospital knew about the study except the main investigator and she had no connection with the antenatal care team. All personal information and study data were stored on the investigator’s computer, which was password protected and only accessible by the investigator. It is worth noting that some of our participants were under age 18. According to the Article 1041 of Civil Law the minimum age of marriage in Iran is 13 for girls and 15 for boys (http://www.ghavanin.ir/detail.asp?id=16686), ratified by *The Expediency Discernment Council* (http://maslahat.ir/DocLib2/Approved Policies/expediency council in noncompatabilities/NC1381/NC- 01-04-1381-NC55.aspx). Thus when a girl becomes a ‘married woman’ even if under age 18, she does not need informed parental consent and she has her own rights to make decisions as a mature individual. Finally we need to clarify that since In Iran this is a very usual practice that married women have their own rights, at present there is no any particular national guidelines to address this for participating in a scientific research.

## Results

### Women’s characteristics

In all 266 eligible pregnant women were approached and 230 women were agreed to participate in the study giving a response rate of 86.5 %. The mean age of women was 26.1 (SD = 4.95), ranging from 15 to 40 years. Thirty-one women (13.5 %) were aged between 15 and 20 years. Overall 149 women (64.8 %) reported intimate partner violence during pregnancy (76 physical and 73 psychological). The women’s characteristics are shown in Table 1.

**Table 1:** The characteristics of the study samples

	Total (*n* = 230)	Non-abused (*n* = 81)	Physical-abused (*n* = 76)	Psychological-abused (*n* = 73)	*p*-value
Age group (No., %)					0.07*
15–20	31 (13.5)	9 (11.1)	11 (14.5)	11 (15.1)	
21–25	87 (37.8)	32 (39.5)	27 (35.5)	28 (38.4)	
26–30	73 (31.7)	34 (42.0)	21 (27.6)	18 (24.7)	
31–40	39 (17.0)	6 (7.4)	17 (22.4)	16 (21.9)	
Mean (SD)	26.0 (4.9)	25.4 (4.0)	26.2 (5.2)	26.6 (5.4)	0.33**
Education (No., %)					0.0005*
Primary	60 (26.1)	11 (13.6)	24 (32.9)	25 (32.9)	
Secondary	157 (68.2)	62 (76.5)	48 (65.8)	47 (61.8)	
Higher	13 (5.7)	8 (9.9)	1 (1.4)	4 (5.3)	
Parity					0.01*
No child	54 (23.5)	17 (21.0)	17 (22.4)	20 (27.4)	
1–2 children	140 (60.9)	59 (72.8)	46 (60.5)	35 (47.9)	
3–5 children	36 (15.7)	5 (6.2)	13 (17.1)	18 (24.7)	
Mean (SD)	1.35 (1.13)	1.2 (0.94)	1.5 (1.33)	1.36 (1.09)	0.26**

* Derived from chi-square (secondary and higher education was treated as one category)

** Derived from one-way analysis of variance

### Quality of life

An independent-samples t-test was conducted to compare SF-36 scales for abused and non-abused pregnant women. There was a significant difference in the scores for abused and non-abused groups on all scales with the exception of bodily pain (see Table 2). For example, abused women reported significantly lower mean scores for physical functioning (Men = 53.9, SD = 23.5) than non-abused women (Mean = 68.7, SD = 22.4); (t = 4.61, df = 228, *p* < 0.001). These results suggest that abuse during pregnancy negatively impacts on women’s physical, social and mental health.

**Table 2:** Comparing quality of life scores between abused and non-abused groups*

	All (*n* = 230)	Non-abused (*n* = 81)	Abused (*n* = 149)		
	Mean (SD)	Mean (SD)	Mean (SD)	t-value	*p*-value**
Physical functioning	59.1 (24.1)	68.7 (22.4)	53.9 (23.5)	4.61	<0.0001
Role physical	31.9 (34.1)	43.2 (39.5)	25.8 (29.2)	3.78	<0.0001
Bodily pain	65.6 (25.9)	70.7 (26.0)	64.2 (26.3)	1.08	0.27
General health	64.2 (22.8)	69.5 (21.9)	61.4 (22.9)	2.59	0.01
Vitality	47.7 (22.2)	55.6 (21.6)	43.5 (21.5)	4.06	<0.0001
Social functioning	61.0 (25.0)	70.5 (23.4)	55.8 (24.4)	4.39	<0.0001
Role emotional	29.2 (37.0)	46.0 (42.6)	20.1 (29.9)	5.37	<0.0001
Mental health	58.2 (20.4)	63.5 (19.3)	55.3 (20.4)	2.95	0.003

* Higher scores indicate better health

** Derived from two independent samples t-test

One-way between subjects ANOVAs were conducted to compare the impact of type of abuse - physical, psychological or no abuse - on physical and mental health as measured by the SF-36 subscales. There was a significant main effect for all SF-36 subscales at the *p* < 0.05 level (see Table 3). Post hoc comparisons using the Tukey HSD test indicated that the mean scores for the psychologically abused women were significantly lower than the non-abused women on physical functioning, role physical, general health, vitality, social functioning and mental health. Physically abused women were significantly lower than the psychologically abused women for role physical, bodily pain and general health (see Table 3). However, the psychologically and physically abused women did not differ significantly on physical functioning, vitality, social functioning, role emotional or mental health. Specifically, our results suggest that physical abuse impacted significantly on women’s physical health. Psychological and physical abuse had similar impacts on the social and mental SF-36 scales.

**Table 3:** Comparing quality of life scores among non-abused, physical abused, and psychological abused groups*

	Non-abused (*n* = 81)	Physical-abused (*n* = 76)	Psychological-abused (*n* = 73)		
	Mean (SD)	Mean (SD)	Mean (SD)	F value	*p*-value***
Physical functioning	68.7 (22.4)	53.0 (23.1)	54.8 (24.1)^a^	10.73	<0.0001
Role physical	43.2 (39.5)	21.0 (29.1)	30.8 (28.7)^a^, ^b^	8.86	<0.0001
Bodily pain	70.7 (26.0)	58.0 (25.3)	68.1 (25.0)^b^	5.26	0.006
General health	69.5 (21.9)	57.5 (23.3)	65.1 (22.1)^a^, ^b^	5.54	0.004
Vitality	55.6 (21.6)	46.2 (21.2)	40.6 (21.5)^a^	9.55	<0.0001
Social functioning	70.5 (23.4)	56.0 (25.4)	55.6 (23.5)^a^	9.63	<0.0001
Role emotional	46.0 (42.6)	21.0 (29.1)	19.2 (30.9)^a^	14.45	<0.0001
Mental health	63.5 (19.3)	57.0 (20.1)	53.5 (20.7)^a^	4.93	0.008

* Higher scores indicate better health

** Derived from one-way analysis of variance (ANOVA)

^a^ Post hoc Tukey test comparison of psychologically abused vs. non abused women significant at *p* < 0.05

^b^ Post hoc Tukey test comparison of psychologically abused versus physically abused significant at *p* < 0.05

General health and mental health subscales were dichotomized as higher (better health) or lower (worse health) than the mean scale score to examine the impact of abuse after adjusting for demographic factors associated with poor health. Finally the result obtained from logistic regression analyses showed that after adjusting for age, education and parity the most significant contributing factors to the poor general health was physical violence (OR = 2.13, 95 % CI = 1.05–4.36, *p* = 0.03) and to the poor mental health was psychological violence (OR = 1.89, 95 % CI = 1.09–3.84, *p* = 0.04). The results are shown in Table 4.

**Table 4:** The results obtained from logistic regression analysis for the poor general and mental health

	No. (%)^a^	Adjusted OR	95 % CI	*p*-value
General health				
Age				
15–20	18 (58.0)	1.80	0.66–4.92	0.25
21–25	44 (50.5)	1.61	0.64–4.03	0.31
26–30	20 (27.3)	1.60	0.47–5.45	0.44
31–40	10 (25.6)	1.0 (ref)		
Education				
Higher	4 (30.7)	1.0 (ref)		
Secondary	60 (38.2)	1.01	0.52–1.96	0.97
Primary	28 (46.6)	1.52	0.71–3.24	0.27
Parity				
No child	31 (57.4)	1.37	0.25–1.60	0.33
1–2 children	49 (35.0)	1.11	0.26–2.51	0.71
3–5 children	12 (33.3)	1.0 (ref)		
Violence				
None	25 (27.2)	1.0 (ref)		
Physical	38 (41.3)	2.13	1.05–4.36	0.03
Psychological	29 (31.5)	1.24	0.62–2.48	0.53
Mental health				
Age				
15–20	20 (64.5)	1.62	0.49–5.32	0.42
21–25	39 (44.8)	1.52	0.62–3.68	0.35
26–30	24 (32.8)	1.04	0.39–2.74	0.94
31–40	12 (30.7)	1.0 (ref)		
Education				
Higher	5 (38.4)	1.0 (ref)		
Secondary	61 (38.8)	1.07	0.55–2.08	0.83
Primary	29 (48.3)	1.28	0.61–2.73	0.42
Parity				
No child	28 (51.9)	1.35	0.32–2.15	0.23
1–2 children	55 (39.2)	1.11	0.29–1.32	0.58
3–5 children	12 (33.3)	1.0 (ref)		
Violence				
None	26 (32.1)	1.0 (ref)		
Physical	31. (40.8)	1.27	0.64–2.51	0.49
Psychological	38 (52.1)	1.89	1.09–3.84	0.04

^a^ Number (row %) for poor physical health (*n* = 92) and poor mental health (*n* = 95)

## Discussion

We found that a considerable number of pregnant women were exposed to intimate partner violence. The findings confirm previous observations from Iran [7–9] that are alarming and needs urgent attention for providing support services for victims. Consideration of effective ways to prevent and address such violence against women is important, not only for women’s health, but also that for the health of their unborn baby and any other children in the family. A recent study from Iran showed that women with lower education and living in low income households reported more intimate partner violence during pregnancy than well-educated and affluent women [32]. Such findings suggest that providing equal opportunity for women by legal and official means should be considered seriously in Iran and countries with similar conditions.

This study investigated quality of life in three groups of pregnant women and the findings indicated that psychological violence could have significant association with women’s quality of life as much as physical violence. This study highlights the fact that the physical violence impacts on women’s lives, but psychological abuse also significantly affects pregnant women’s physical and mental health. It is argued that physical abuse is an apparent phenomenon [1] but psychological violence might not be detected very easily. A recent publication on intimate partner violence before and during pregnancy found that 14.9 % of women experienced psychological abuse while only 2.5 % of women reported physical abuse [33]. Thus it seems that screening for psychological violence against pregnant women should be integrated into antenatal care services to support the health and well being of women and their families. As suggested health care providers are urged to identify those women at risk so that antenatal care can be tailored to best support optimal maternal and neonatal outcomes [34]. However, one might argue that pregnant women are usually reluctant to disclose intimate partner violence to the healthcare team. Fortunately a recent review on screening women for intimate partner violence in healthcare settings indicated that pregnant women in antenatal settings may be more likely to disclose intimate partner violence when screened [35].

There have been several studies on the impact of domestic violence on different aspects of women’s life during pregnancy [23–26, 36]. Thus it is argued that deeper understanding is needed to indicate the actual impact of this complex matter. For instance a qualitative study found that ‘struggling to survive for the sake of the unborn baby’ was the main concern of women who were exposed to intimate partner violence during pregnancy [37] or a study on midwives experiences reported that ‘it is difficult to recognize domestic violence’ because of a limited knowledge of the most common signs and symptoms of violence, a lack of training, cultural taboos, and the women׳s unwillingness to disclose abuse [38]. We feel similar situation exist in Iran and antenatal care team usually do not ask pregnant women about domestic violence and even if they do so there is no way to get support for such victims. We believe young, and less educated women are more likely to suffer from intimate partner violence during pregnancy. Therefore we suggest the antenatal care team should take responsibility and make themselves familiar with the issue and at least find ways to support high-risk groups.

### Limitations

The current study showed a significant difference in quality of life between abused groups (physical and psychological) and non-abused group. Yet, the results could not be generalized to all women since this was a descriptive study in nature with a limited sample size and even our exclusion criteria would exclude a large number of abused women as these criteria were closely associated with domestic violence. In addition we used a general questionnaire for measuring quality of life while it seems that more specific measures are required in order to explore the influence of different types of abuse on women’s quality of life and their mental health. Furthermore it is difficult to measure general and mental health if one does not ask about women’s health before pregnancy, especially mental health, as we did not. A new study provides good evidence for the importance of this [39]. Finally, we know that the SF-36 has Physical and Mental Health Component Summary. Unfortunately we did not have access to the SF-36 software to calculate theses and thus we used the general and the mental health subscales instead. This also should be seen as a limitation.

## Conclusions

The findings demonstrated that intimate partner violence have significant association with quality of life in pregnant women. Prevention, and detection of violence against pregnant women need urgent action by primary health care team in order to improve women’s both overall and reproductive health.
